# The Impact of Population Aging and Public Health Support on EU Labor Markets

**DOI:** 10.3390/ijerph17041439

**Published:** 2020-02-24

**Authors:** Mirela Cristea, Gratiela Georgiana Noja, Petru Stefea, Adrian Lucian Sala

**Affiliations:** 1 Department of Finance, Banking and Economic Analysis, Faculty of Economics and Business Administration, University of Craiova, Center for Banking and Financial Research, 13 A I Cuza Street, 200585 Craiova, Romania; 2 Department of Marketing and International Economic Relations, Faculty of Economics and Business Administration, West University of Timisoara, East European Center for Research in Economics and Business, 16 Pestalozzi Street, 300115 Timisoara, Romania; gratiela.noja@e-uvt.ro; 3 Department of Management, Faculty of Economics and Business Administration, West University of Timisoara, 16 Pestalozzi Street, 300115 Timisoara, Romania; petru.stefea@e-uvt.ro; 4 Doctoral School of Economics, Faculty of Economics and Business Administration, University of Craiova, 13 A I Cuza Street, 200585 Craiova, Romania; sala_lucian@yahoo.com

**Keywords:** older workers, labor productivity, labor market policies, health expenditure, econometric procedures, European Union

## Abstract

Population aging and public health expenditure mainly dedicated to older dependent persons present major challenges for the European Union (EU) Member States, with profound implications for their economies and labor markets. Sustainable economic development relies on a well-balanced workforce of young and older people. As this balance shifts in favor of older people, productivity tends to suffer, on the one hand, and the older group demands more from health services, on the other hand. These requisites tend to manifest differently within developed and developing EU countries. This research aimed to assess population aging impacts on labor market coordinates (employment rate, labor productivity), in the framework of several health dimensions (namely, health government expenditure, hospital services, healthy life years, perceived health) and other economic and social factors. The analytical approach consisted of applying structural equation models, Gaussian graphical models, and macroeconometric models (robust regression and panel corrected standard errors) to EU panel data for the years 1995–2017. The results show significant dissimilarities between developed and developing EU countries, suggesting the need for specific policies and strategies for the labor market integration of older people, jointly with public health expenditure, with implications for EU labor market performance.

## 1. Introduction

Health systems in developed and developing countries are in the process of adapting to population aging, due to significant increases in life expectancy, along with a downturn in birth rates [1]. Wide-ranging population aging is commonly associated with many consequences, such as increased costs for households, strains on public finances and healthcare providers and decreasing economic growth [2,3,4,5].

As demographic structures shift towards an aging population, greater pressure will be placed on the population of working age, particularly those aged between 55 and 64 years [6,7]. In most European Union (EU) Member States (MS), healthcare costs are covered from both private and public sources, while the public ones are financed by contributions collected from the working age population. Thus, as the share of older recipients of services increases, so will their dependency on the working population, since the number of employed individuals will remain steady or decline. According to forecasts, the old age dependency ratio “has risen to 29.6% in 2016 and is projected to rise further, in particular up to 2050, and eventually reach 51.2% in 2070” [8] (p. 3), leading to decreased social security contributions collected from the population of working age. Thus, population aging applies high pressure on policy-makers, with critical challenges going forward.

In this regard, a series of policies and approaches have been implemented to ease the effects of aging, jointly with public healthcare service enhancement, and also to assure better utilization of existing infrastructure and human capital [9,10,11,12]. The EU-28 MS, on an individual basis, have made adjustments to healthcare to curb costs and transfer primary care from hospitals to local community centers, with promising results [8,10]. The focus within EU countries is on healthy and active aging, since the share of the population aged 65 and over within the EU grew from 14.9% in 1996 to 19.2% in 2016, with projections of 29% in 2070 [8]. Based on these projections “Europe will be most affected by population ageing” [13] (p. 7).

When contemplating these challenges, it can be argued that, in the absence of solid and versatile policies, a major restructuring of economic activity in both the public and private sectors might bring the end of the “welfare state” [14]. However, the majority of studies do not seem to support the argument that population aging will cause uncontrollable public sector expenditure [15,16,17].

Based on these facts, the links between population aging and increases in healthcare costs [18,19,20], as well as their implications for labor productivity [21,22,23], have been intensively debated and documented in the scientific literature, but, to the best of our knowledge, comparisons of the EU’s developed and developing countries have not been undertaken.

Based on these underpinnings, the general objective of our paper is to assess the effects of population aging and public health expenditure on labor market performance within the EU-28 MS, with a detailed analysis of the EU-15 (developed countries) and the EU-13 (developing countries) MS. We target four specific research guidelines and associated working hypotheses: respectively, the implications of the employment rate of the population aged 55–64 for labor productivity, the implications of health expenditure on aging coordinates (birth rate and life expectancy) and the health of older people (overall population health perception), as well as the overall (direct, indirect, total) joint implications of health dimensions (namely, health government expenditure, hospital services, healthy life years, perceived health) and aging for labor productivity. Our research is configured to enrich the literature with an updated, complex, and integrative assessment of the interlinkages between the aging phenomenon, health features, and labor productivity, by applying different econometric methods and techniques to ensure robust and accurate results that provide the groundings for our conclusions and policy recommendations. This research complements diverse strands of thought and contributes to increasing awareness concerning the fundamental importance of the aging process, through its multidimensional facets (social, psychological, political, and economic). Furthermore, it identifies the essential mechanisms, policies, and strategies that need to be designed and implemented in a tailored framework within the EU to support active and healthy aging, with benefit spillovers for the wellbeing of older people and the welfare of society as a whole.

We chose to compare these two panels of EU countries (EU-15 and EU-13), since there are specific dissimilarities between them, associated with homogeneous characteristics inside these groups, as regards the aging population (captured by the active aging index—AAI) and economic development relationship, as the UNECE/European Commission [24] has outlined. A comparative analysis within the EU MS between developed (EU-15) and developing (EU-13) countries provides a clearer picture of the tailored specific strategies that can be adopted for each group of countries, and to rethink the approach of covering the healthcare needs of older individuals. The dataset utilized in the analysis covers the period 1995–2017, compiled based on the availability of representative aging indicators, and health- and labor market-specific variables. The methodology applied consists of three advanced econometric procedures: (1) Structural equation modeling (SEM); (2) Gaussian graphical models (GGMs); and (3) macroeconometric models (robust regression (RREG) and panel-corrected standard errors (PCSE)), each of them on two distinct panels (the EU-13 and the EU-15 groups of countries), to synergistically assess the influences of population aging on labor productivity, shaped by the health coordinates (namely, health government expenditure, hospital services, healthy life years, perceived health).

The rest of the paper is organized as follows. Section 2 elaborates a theoretical framework, where we summarize the most relevant studies on how population aging and healthcare expenditures tend to affect associated labor market outcomes, under digital transformations, associated with strategies and policies advanced by diverse strands of thought. Section 3 describes the data and methodology applied. Section 4 reports the results and provides a discussion of their implications. Section 5 offers final concluding remarks. Additional information and empirical evidence are provided as appendices.

## 2. Theoretical Framework

The link between healthcare spending and its effects on population aging, in relation to economic growth or labor market outcomes, has received a great deal of attention in the scientific literature, with an emphasis on national and regional policies and strategies employed to address changes in expenditure patterns.

### 2.1. Health and Aging

Population aging has led to consideration of the potential for increased medical costs and long-term care services for the older segment of the population, and to the imbalance between those of working age and the elderly through shifting dependency ratios. As the elderly dependency ratio increases, it is assumed that individuals will become more dependent on social services, whilst healthcare needs will increase due to conditions that accompany old age [25,26].

Healthcare expenditure at the level of the EU-28 is expected to increase between 2016 and 2070 due to changes in population structure in both the EU-15 and EU-13 MS [17]. Anticipated increases in expenditure will be driven by gains in longevity that lack increases in the quality of health of individuals, causing an increase in demand for healthcare services [18].

Withal, increases in health expectancy can be credited to a great degree to education, where, on average, “people with a low level of education can expect to live six years less than those with a high level of education” [10] (p. 12). These findings are explained by the fact that less educated people are less likely to participate in preventive healthcare, as Bremer et al. [27] found in the case of Germany. Furthermore, gains in life expectancy in EU-28 MS slowed, on average, between 2011 and 2018 by nearly 50% when compared to previous periods, mainly due to slower improvements and medical breakthroughs in terms of the management of diseases of the circulatory system and treatment of conditions associated with the later stages of life [28,29,30].

Several studies also put together aging and health in conjunction with other demographic factors (such as mortality, birth rate, people mobility), no education (“illiteracy rate”), the residence environment (“urbanization”), and industry sectors (main productive ones) [31] (pp. 6–7). As regards the implications of health and education upon aging, Yang et al. [31], by applying multifactorial econometric models for 2000 and 2010 in China, found favorable impacts induced by education and healthcare conditions on the evolution of the aging pattern, although at a decreasing rate over time.

Forecasts of healthcare expenditure by age group are driven, for the most part, by population aging, and are dependent on two categories of factors: firstly, changes in spending patterns between younger and older generations; and, secondly, the size of the elderly share of the total population. In the first category, governments can intervene to appease conflicts between younger and older cohorts through spending policies [17].

Regarding the link between population aging and increases in healthcare costs, besides age, a series of other factors shape the level of healthcare expenditure within developed and developing nations, as follows [8,10]: the size and age structure of a certain population; quality of life; access to basic hygiene and nutrition; the health of the elderly and elderly share of the total population; the “cost of dying”; the size of the national economy as seen through gross domestic product (GDP) and the size of GDP per capita; personal choices (alcohol and cigarette consumption); environmental factors (i.e., air quality, electromagnetic pollution); preventive policies through primary care physicians and national awareness programs; new technological breakthroughs that help to cure existing conditions and innovations that optimize costs; the average cost of treatment in private versus public care facilities; the gross yearly cost of staffing hospitals and other healthcare facilities at a national level; the gross yearly cost associated with running and suppling hospitals and other healthcare facilities; and the total amount paid by individuals to public and private health insurance schemes.

### 2.2. Health, Aging, and Welfare/Labor Market

Aging populations are rapidly transforming economies by shifting demand for both goods and services to other sectors of the economy, due to the changing needs of older cohorts. As regards the effects of aging on labor productivity, it is argued that “ageing may represent a drag on labor productivity growth” [23] (pp. 90–91) due to the following factors: technological achievements that have led to a reduced share of workers involved in “manual labor (blue collar occupations)”, where those aged 55–64 are overrepresented; and reduced cognitive skills of older workers, skills that “are becoming increasingly important in the workplace”. Favorable impacts of aging upon labor productivity could be achieved by [23]: higher health expenditure allocated to support the active participation of the population aged 55–64 in the labor market, which would lead to better health conditions and increased productivity; the increasing trend in educational attainment of the workforce aged 55–64 would lead to better performances, since “more educated workers are better able to adapt to offset any negative effect of ageing on productivity”; and the flexibility of the employers in programming work, with “opportunities for part-time work, contract work, and telecommuting” would meet the needs of some older workers. In order to increase the labor market integration of older workers, Taylor [32] (p. 24) offers four distinctive workplace solutions: “Learning, training and development, flexible working practices and the modernization of work, workplace design and health promotion, changing attitudes within the organization”.

From a different perspective, Bussolo et al. [26] examined health, aging, and macroeconomic effects in Europe and Central Asia and argued that aging would lead to an increase in labor productivity due to the extension of working age as a result of life expectancy rising. When examining how this phenomenon influences policies within the EU-28 MS, it becomes obvious that, as some researchers have pointed out, “gloom and doom” predictions are frequently misplaced, and population aging does not present as heavy a social and economic burden as once considered [19,33,34]. On the contrary, aging populations have favorable impacts on labor productivity, as evidenced in multiple studies [10,20,35,36,37,38]. Aging generates increases in the needs of old cohorts that are not as high as originally thought, and older individuals in good health tend to contribute long after retiring by providing compensated and uncompensated labor. Thereby, these findings demonstrate that, particularly in developed countries, older individuals contribute to society and to economic growth even after retirement. Hence, there is a keen need to strengthen the literature with new evidence on how to encompass the health and skills of older people in light of the aging phenomenon, and to support their proper integration in the labor market so as to enhance their labor productivity, with benefit spillovers for sustainable economic development. This study contributes to enlarging the discussion and de-gaps many of these essentials.

Furthermore, older people who have left the workforce continue to contribute through taxation and other forms of income redistribution, helping to sustain public sector expenditures [10]. Moreover, many older people have accumulated assets (real estate, financial assets, deposits) that contribute to welfare/economic growth and help sustain spending patterns, where a significant amount of expenses comes from income sources other than transfers from social security and pension schemes [8,39].

People who exit the labor force after the age of 64 or sooner, or who benefit from transfers from the public sector, are generally defined as “dependent”, with small contributions to society and to economic growth [34]. However, the choice to continue to work can be made if health permits this, with positive benefits for individuals and society. Opting to continue to work depends also on policies implemented at a national level that encourage individuals to work past retirement age, on the performance of pension systems, on how employers view older workers, on how the productivity of older employees is perceived, and also on the flexibility of working hours in some circumstances [11,40,41]. Healthy older people tend to contribute through direct or indirect channels to welfare by undertaking voluntary work or by offering informal caregiving, “raising the future generations” [42] (p. 15). Thus, the term “silver economy” has been coined when referring to the advantages that an aging population brings to communities [34].

### 2.3. Implications of the “Digital Era”

A greater emphasis needs to be placed on adopting new technologies and changing approaches with regard to care services offered to the older population, since “both health spending and technological progress are a potential source of better welfare outcomes in terms of longevity” [43] (p. 8). Population aging induces growing costs in healthcare services, due to an increase in the utilization of age-related procedures and treatments that are pushing up costs of long-term care, which are expected to grow at a faster pace than other healthcare needs. In addition, improving datasets on age groups and conditions and integrating them in an easily accessible, secure network will ensure agility in providing services [44].

As a consequence, a myriad of studies put the spotlight on increasing research and development (R&D) in the health field, since healthy individuals are more productive and generate competitiveness [45], raising the standard of living for all [46,47].

Likewise, emerging technologies have helped to extend life expectancy, but without providing a permanent cure for common ailments. Thus, individuals tend to live longer lives, but with acute chronic conditions, adding to healthcare costs [44]. This will most likely be the case in the future, as demand for the adoption of new more expensive technologies becomes keenly requested by cohorts [48,49].

Furthermore, technological innovations entail the need for consideration of the role of active labor market policies (ALMPs), “aimed at reskilling the workers and at making them fit for the coming digital economy as pivotal issues” [50] (p. 9), especially for the older workforce.

### 2.4. Policies and Strategies

The focus of policy-makers is converging on *healthy and active aging*, with the aim of increasing the economic contribution of older people in order to help the transition of developed and developing nations to a “silver economy” status or “golden age” [8,26,34,51]. *Active aging* was put into EU strategy in 2012 [52] by setting its coordinates, together with specific policies for all MS, in an effort to increase opportunities for older people to continue to work and contribute to society in other ways and stay healthy for longer. Based on a literature review, Álvarez-García et al. [53] (p. 4) conclude that “the active ageing concept is currently based on four main pillars: participation, health, security and lifelong learning”. According to the European Commission [9], active aging refers to “helping people stay in charge of their own lives for as long as possible as they age and, where possible, to contribute to the economy and society” or “growing older in good health” [24] (p. 4). With these underpinnings, a composite instrument was framed for the EU-28 MS in order to assess and reveal the “active and healthy life of older people” [24] (p. 5), namely “the Active Ageing Index (AAI)” [54], which was measured for the years 2010, 2012, and 2014. The results revealed profound dissimilarities among developed and developing EU countries [24].

The *healthy aging* concept was included in the active aging framework [55], and was reinforced by the strategy of the World Health Organization (WHO) [12]. Healthy aging represents “the process of developing and maintaining the functional ability that enables wellbeing in older age”, in order to achieve sustainable development goals (SDGs), aging being “relevant to 15 of the 17 Goals” [12] (p. 4). The WHO’s strategy has five objectives, focused on two actions: “Five years of evidence-based action to maximize functional ability that reaches every person; and, by 2020, establish evidence and partnerships necessary to support a Decade of Healthy Ageing from 2020 to 2030” [12] (p. 6).

Population aging will require a transformation of healthcare service providers, requiring new approaches to streamline healthcare services and optimize costs. A solution could be to transfer manageable cases from hospitals to local “people-center” care providers in the community [10,56]. These are the long-term guidelines in the EU countries where decreases in hospital capacity have been implemented, along with a reduction in the average number of days when patients are hospitalized.

Increasing the efficiency of healthcare spending within both developed and developing countries represents a top priority, since “up to one-fifth of health spending is wasteful and could be reallocated to better use” [10] (p. 4). The main causes are linked to healthcare professionals administering unnecessary treatments or medical tests, and where the utilization of cheaper treatments would produce the same curative effects as more expensive ones. Thus, reducing unnecessary spending without an effect on the quality of healthcare offered could prove a viable objective for the long-term sustainability of the healthcare system.

In terms of demographic aging, there is a wide gap between developed and developing countries, with different speeds among them [57,58], since developed countries are confronted to a greater extent with increased old dependency ratios (e.g., Italy, Portugal, Greece, Finland, Sweden, and Germany) [30]. Notable differences between developing and developed countries in terms of the implications of aging for health services have been identified by Lloyd-Sherlock [59], who states that, given the complexity and widespread of synergy between aging and health expenditure, less developed countries face a gap as regards the specific policies adopted. Moreover, Lloyd-Sherlock [59] (p. 887) notes that “patterns of ageing and their implications for policy are highly complex and variable”, shaped by each country’s environment (social, political, and economic) and, as a consequence, it would not be relevant to investigate them on a global perspective. Referring to differences between countries with regard to aging and labor productivity, Börsch-Supan [57] (p. 1) stressed that “due to the globalization of our economies, no study of aging can disregard these differential changes”.

For the purpose of optimizing resource utilization, a series of measures have been suggested by the Organisation for Economic Co-operation and Development (OECD) [10]: (1) Guaranteeing a high exchange rate between invested capital and products and services provided, procured and quoted process of pharmaceutical products using the “Health Technology Assessment” framework; (2) increasing possible cumulated funds from generics and biosimilars; (3) raising standards for more targeted prescriptions; and (4) increasing devotion on behalf of patients through awareness programs.

In order to sustain labor markets against a background of population aging the OECD [11] has proposed specific actions for policy-makers, social organizations, and individuals. Policy-makers need to provide incentives for the older workforce to remain in the labor market “by removing penalties to later retirement and providing more flexible work/retirement options”. The social partners need remove age discrimination from the labor market by employers “by promoting good management practices for age-diverse workplaces”, and offer training opportunities for older workers.

Accordingly, after reviewing the scientific literature, we infer that the connection between population aging, health, and labor productivity has been widely explored, being opened to debate, since the EU is highly affected by population aging and needs in-depth and continuous assessments targeted at specific development levels of MS. We summarize that, although there are various approaches as regards the conjunction between aging and health upon productivity, few studies have investigated the distinct groups (i.e., developed and developing) of countries within the EU; aging is increasing healthcare expenditure, there being a direct connection between aging and health; most studies suggest that the impact of aging upon productivity in the long run is unfavorable; healthy older individuals can actually enhance productivity through direct or indirect labor force participation (active and healthy aging); and essential aspects like education and lifelong learning, R&D and innovation expenditure, and active labor market policies, need to be enclosed by policies and strategies.

## 3. Data and Methodology 

Based on the literature review, we included educational components [10] and R&D expenditure [28,29,30] among the specific variables comprising our models, alongside aging, health, and labor market representative indicators.

The dataset was divided into two panels of EU Member States, the developed EU-15 countries and developing EU-13 MS. The analyzed period covered the years 1995–2017 and encompassed specific variables on aging representative indicators, health measurements, and labor market and other specific socio-economic indicators, as follows (Table A1):

- *Aging representative indicators*: Employment rate, 55–64-year-old group (% of total population) (*ER_55_64*); life expectancy at birth, total population (years) (*LE*); crude birth rate (number of live births per 1000 people) (*BR*).
- *Health indicators*: Health government expenditure (% of GDP) (*HGE*); hospital services (% of GDP) (*HS*); healthy life years in absolute value at 65—females (years) (*HLY_F*); healthy life years in absolute value at 65—males (years) (*HLY_M*); share of people (aged 16+) with good or very good perceived health (%) (*PGPH*), as a targeted indicator of the Sustainable Development Goals (SDG), namely, SDG3 “Good health and wellbeing”, which “has been found to be a good predictor of people’s future health care use” [30].
- *Labor market and other specific indicators*: Labor productivity per person employed and hours worked (%, EU-28 = 100) (*LP*); active labor market policies (% of GDP) (*ALMP*); passive labor market policies (% of GDP) (*PLMP*); annual net earnings (purchasing power standard—PPS) (*EARN*); tertiary education level, 30–34-year old group (% of the population aged 30–34) (*TE_30_34*); population with secondary, upper, post-secondary, and tertiary education for 15–64-year-old group (levels 3–8) (% of 15–64 years) (*EDU*); total R&D expenditures (% of GDP) (*GERD*).

The data were extracted from the European Commission—Directorate-General for Employment, Social Affairs & Inclusion [60] for the active and passive labor market policies, and Eurostat [30] for all other variables.

**Figure 1:**
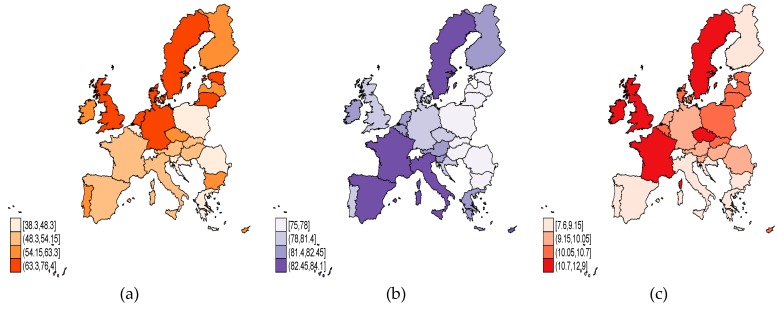
Aging representative indicators, European Union (EU)-28, 2017: (**a**) Employment rate, 55–64 years *(ER_55_64)*; (**b**) life expectancy *(LE)*; (**c**) birth rate (*BR).* Source: Own process in Stata.

As regards the *aging representative indicators* at the level of EU MS in 2017 (Figure 1), we can outline that there are distinctive results for the EU-15 and EU-13 panels, more emphasized for the former. The employment rate of people aged 55–64 years (*ER_55_64*) (Figure 1a) is at its highest levels in the Nordic States (Sweden, Denmark), alongside the United Kingdom (UK), the Netherlands, and Germany from the EU-15 group, but also Estonia and Lithuania from the EU-13 panel. Life expectancy (*LE*) (Figure 1b) is much higher across the EU-15 countries—namely, Italy, Spain, France, Luxembourg, and Sweden (over 82 years, and increasing)—than the EU-13 developing MS, except for Malta and Cyprus, where it also exceeds 82 years. The highest concentration of population aging is registered in Italy, where, during the period 1995–2017 [30], the birth rate (*BR*) continuously slowed down (from 9.2 in 1995 to 7.6 in 2017 live births per 1000 people), but also in Spain, Portugal, Finland, and Greece being at the lowest level from the EU-15 (Figure 1c). On the ceaseless decreasing trend of the birth rate, the most prominent values within the EU-28 were registered in 2017 by Ireland (12.9 live births per 1000 people), France, the UK, and Sweden from the EU-15 panel, and the Czech Republic from the EU-13 group.

**Figure 2:**
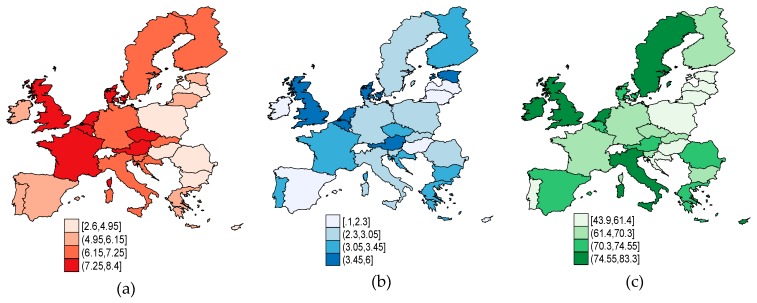
Health illustrative indicators, EU-28, 2017: (**a**) Health government expenditure (*HGE*); (**b**) hospital services (*HS*); (**c**) people (aged 16+) with good or very good perceived health (*PGPH*). Source: Own process in Stata.

Health dimensions within the EU-28 in 2017 (Figure 2) reveal the following: The most significant expenditure on health by governments in terms of GDP (*HGE*) occurs in France, the UK, Denmark, Belgium, the Netherlands, and Austria, but also in the Czech Republic (Figure 2a). The most substantial hospital services (*HS*) are allocated to the same countries as *HGE* (slightly less in France) from the EU-15, and Estonia from the EU-13 panel (Figure 2b). The highest proportion of people (aged 16+) with good or very good perceived health (*PGPH*) (Figure 2c) is in Ireland (over 83%), followed by Italy, Sweden, the UK, and the Netherlands from the EU-15, but also Cyprus and Malta from the EU-13, with over 75% of the population aged 16+. The lowest perceived health from the EU-28 was in Lithuania (43.9%). Age-related expenses for both males and females by age as percentage (%) of GDP per capita tend to increase for individuals aged 50+ at a faster pace, as the aging process progresses for both sexes [17].

**Figure 3:**
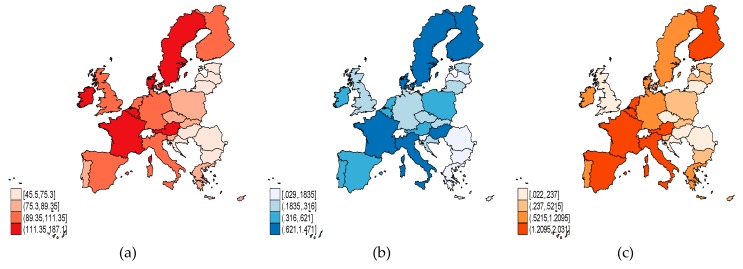
Labor market-specific indicators, EU-28, 2017: (**a**) Labor productivity (*LP*); (**b**) active labor market policies (*ALMP*); (**c**) passive labor market policies (*PLMP*). Source: Own process in Stata.

As regards the labor market-specific indicators (Figure 3), we can see that in 2017, a higher level of labor productivity (*LP*) (Figure 3a) was observed in countries with a significant allocation from GDP of active and passive labor market policies (Figure 3b,c). The highest *LP* in relation to the EU-28 average occurred in Ireland (187.1%), and the lowest was in Bulgaria (45.5%).

For a suitable comparison between EU countries and to manage the stationarity of the indicators, firstly, the dataset was processed through the logarithm procedure. Then, the methodology used consisted of structural equation modeling (SEM), which was applied for the two distinctive panels, EU-13 and EU-15. SEM represents an integrative procedure that appraises overall interlinkages among considered variables, direct, indirect, and total. SEM models are configured through the general system of Equation (1).

(1) $$\left\{ \begin{array}{l} b_{11}y_{2t}+\ldots +b_{1m}y_{mt}+c_{11}x_{1t}+\ldots +c_{1n}x_{nt}=\varepsilon_{1t}\\ b_{21}y_{2t}+\ldots +b_{2m}y_{mt}+c_{21}x_{1t}+\ldots +c_{2n}x_{nt}=\varepsilon_{2t}\\ \ldots \ldots \ldots \\ b_{m1}y_{mt}+\ldots +b_{mm}y_{mt}+c_{m1}x_{nt}+\ldots +c_{mn}x_{nt}=\varepsilon_{mt} \end{array}\right.$$

where *t* is the number of observed time periods, *b_ij_* represents the *y_ij_* endogenous variable’s parameters, *c_ij_* are the *x_ij_* exogenous variable’s parameters, *i* = 1, …, *m*, *j* = 1, …, *n*, and ε comprises the error term (residuals) [61].

**Figure 4:**
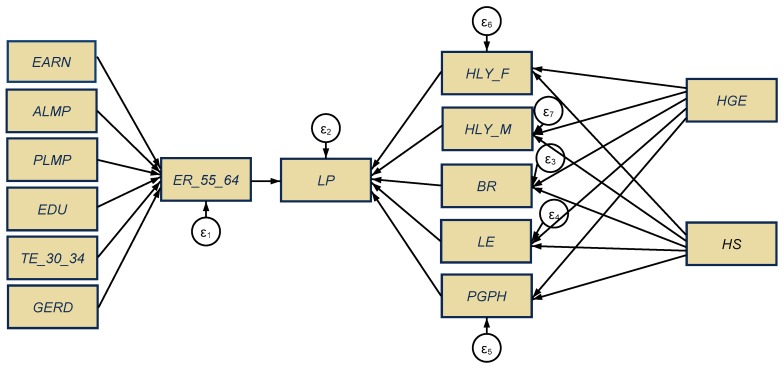
General configuration of structural equation modeling (SEM). Source: Own contribution in Stata.

For our research endeavor, SEM (Figure 4) was utilized to assess *the direct influence* of the aging representative and health variables on labor productivity (*LP*) as follows: The employment rate for the 55–64-year-old group (*ER_55_64*), on the one hand, and the dimensions of aging population (*BR* and *LE*), healthy conditions of people aged 65+ (*HLY_F* and *HLY_M*) and population health perception (*PGPH*), on the other hand.

These influences are appraised under the cumulative effects of two main groups of variables (*indirect influences* upon labor productivity), firstly, *representative factors of labor markets,* which can influence the 55–64 years of age working people, such as the earnings obtained on the labor market (*EARN*), labor market policies, active and passive (*ALMP, PLMP*), educational component for the 15–64-year-old group (*EDU*) and tertiary education for 30–34-year-olds (*TE_30_34*), and R&D expenditure (*GERD*). The other group of indirect variables comprises *health expenditure*, namely, health government expenditures (*HGE)* and hospital services (*HS*) that can influence the aging dimensions (*BR* and *LE*) and people’s health conditions (65+ years, females and males) and perceptions (*HLY_F*, *HLY_M*, respectively, *PGPH*).

In our own research, we have considered and explored the impact that earnings increases can have on labor productivity, since higher wages can spur productivity by acting as an incentive and encouraging employees to engage in productivity enhancing activities [62]. In this frame, the so-called “efficiency wages”, namely wages that are above the market rate, are a key motivation for employees, that provide higher effort levels in response to higher earnings. The employment rate of persons aged 55–64 years was also considered to capture the mechanisms of integration of older people within the labor market.

The research endeavor was also based on several Gaussian graphical models (GGMs) configured through partial correlation (PCOR) and extended Bayesian information criterion (EBIC) with graphical least absolute shrinkage and selection operator (LASSO). These types of models “with sparsity in the inverse covariance matrix are of significant interest in many modern applications” and “indeed lead to better model selection properties in practical relevant settings” [63] (p. 1). Hence, GGMs allow us to further observe the interconnections between all considered processes/coordinates in light of a network setting.

Based on the literature underpinnings and our own methodological endeavor, we tested the following hypotheses (H):

- H_1_: There are significant implications of the employment rate of the population aged 55–64 for labor productivity, more emphasized for developed countries (EU-15) than developing ones (EU-13);
- H_2_: There are significant implications of health expenditure (health government expenditure and hospital services) upon aging coordinates (birth rate and life expectancy) in both EU-15 and EU-13 panels, more prominent for developed countries (EU-15) than developing ones (EU-13);
- H_3:_ There are substantial impacts of health expenditure (health government expenditure and hospital services) on older people’s health conditions and overall health perceptions, both for the EU-15 and EU-13 panels;
- H_4_: There are overall (direct, indirect, total) significant implications of health dimensions and aging upon labor productivity, both for the EU-15 and EU-13 panels.

In order to reinforce SEM and GGM results, each of the above-stated working hypotheses was tested as well by the use of macroeconometric models (simple and multiple regression models), at the level of each distinctive panel, EU-13 and EU-15, during the 1995–2017 timespan. These models were processed through two estimation procedures, namely, robust regression (RREG), based on Cook’s distance (D), Huber, and biweights iterations, and panel-corrected standard errors (PCSE). Robust regression detaches the outliers or high leverage data points in the sample (hence the influential points are dropped), based on Cook’s distance (Cook’s D) and two types of weights on the iteration process (Huber and biweight weighting). At the same time, PCSE accounts for possible deviations in terms of cross-section contemporaneous correlation, as well as heteroskedasticity, thus providing accurate and robust estimates.

The general configuration of the macroeconometric models, deployed to test each working hypothesis, is presented in Equations (2)–(9):

For H_1_:

(2) $$\log\_LP=\delta +\beta_{1}\log\_ER\_55\_64+\theta_{i}+\lambda_{t}+\varepsilon$$

For H_2_ and H_3_:

(3) $$\log\_HLY\_F=\delta +\beta_{1}\log\_HGE+\beta_{2}\log\_HS+\theta_{i}+\lambda_{t}+\varepsilon$$

(4) $$\log\_HLY\_M=\delta +\beta_{1}\log\_HGE+\beta_{2}\log\_HS+\theta_{i}+\lambda_{t}+\varepsilon$$

(5) $$\log\_BR=\delta +\beta_{1}\log\_HGE+\beta_{2}\log\_HS+\theta_{i}+\lambda_{t}+\varepsilon$$

(6) $$\log\_LE=\delta +\beta_{1}\log\_HGE+\beta_{2}\log\_HS+\theta_{i}+\lambda_{t}+\varepsilon$$

(7) $$\log\_PGPH=\delta +\beta_{1}\log\_HGE+\beta_{2}\log\_HS+\theta_{i}+\lambda_{t}+\varepsilon$$

For H_4_:

(8) $$\log\_LP=\delta +\beta_{1}\log\_ER\_55\_64+\theta_{i}+\lambda_{t}+\varepsilon$$

(9) $$\begin{array}{l} \log\_LP=\delta +\beta_{1}\log\_HGE+\beta_{2}\log\_HS+\beta_{3}\log\_HLY\_F+\beta_{4}\log\_HLY\_M+\beta_{5}\log\_BR+\\ +\beta_{6}\log\_LE+\beta_{7}\log\_PGPH+\theta_{i}+\lambda_{t}+\varepsilon \end{array}$$

where δ and β are the parameters that need to be estimated, ε is a stochastic element, and $\theta_{i}$ and $\lambda_{t}$ are variables that capture the country and time effects.

## 4. Results and Discussion

### 4.1. Results of the Structural Equation Model (SEM)

**Figure 5:**
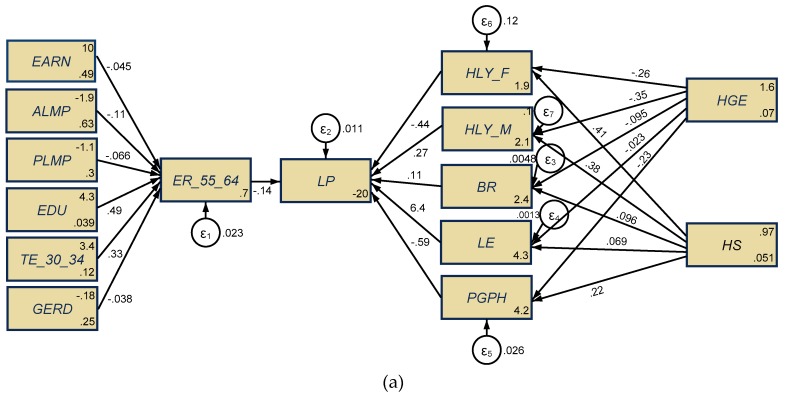

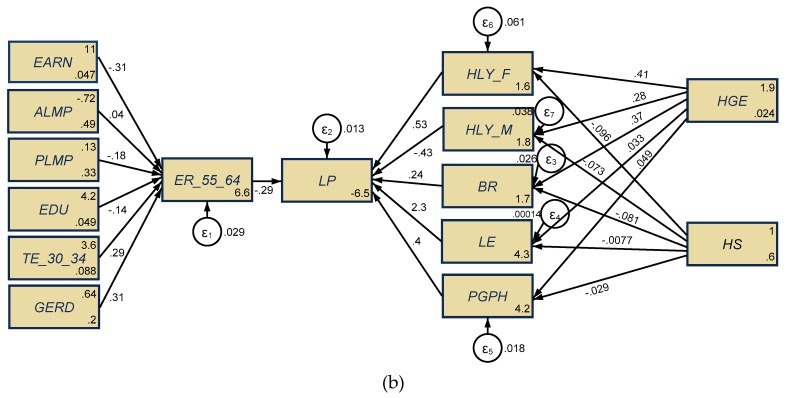
Results of structural equation modeling (SEM) for the EU-13 (**a**) and EU-15 (**b**) panels, 1995–2017. Source: Own contribution in Stata.

We applied the SEM technique for each panel of the EU-28 MS, namely, developing countries, new EU-13 MS, and developed countries, old EU-15 (we included the UK, since the analyzed period was 1995–2017, and the final stage of Brexit has yet to take place). The estimations are graphically represented in Figure 5, while the empirical results on total effects of all considered variables are presented in the Appendix A (Table A2). We applied several tests on the SEM models deployed in order to ensure the validity of the results obtained (also detailed in Table A3, Table A4 and Table A5).

In the case of the EU-13 panel (Figure 5a), we can observe that *LP* is unfavorably influenced, on the one hand, by *ER_55_64* (a coefficient of −0.144, statistically significant, *p* < 0.01), as also stated by [58] and [23]. These outcomes are under the indirect positive influence of only the educational components (*EDU* and *TE_30_34*; both of them are significant from a statistical point of view, *p* < 0.01, more for *EDU* of people aged 15–64 than tertiary education within the 30–34-years-old group), being negatively affected under the other selected variables. Thus, the highest negative influence is the case of *ALMP* (–0.11, significant, *p* < 0.01), followed by *PLMP* (–0.066, statistically significant, *p* < 0.05) and *EARN* (−0.045, statistically significant, *p* < 0.05). In addition, *GERD* also exerts a negative influence on *ER_55_64*, although it is not statistically significant.

Under the indirect influences of health expenditure, *HGE* and *HS,* labor productivity is positively influenced by *LE* (a coefficient of 6.4, statistically significant, *p* < 0.001), *HLY_M* (a coefficient of 0.27, statistically significant, *p* < 0.05), and *BR*, although not statistically significant. The other variables, *HLY_F* and *PGPH*, negatively influence the *LP* within the EU-13 panel (both of them are statistically significant, *p* < 0.001). As regards *HGE,* the results show that government health expenditure is not enough to ensure healthy conditions for people of both genders aged 65+ in terms of good or very good perceived health. Moreover, *HGE* does not seem to encourage the birth rate or sustain life expectancy increases (since the coefficients for all these components are negative and statistically significant). At the same time, the most emphasized unfavorable effects of *HGE* are the health conditions as perceived by the population (*PGPH*) (a coefficient of 0.23, statistically significant, *p* < 0.001), with overflow implications upon *LP*. As for *HS,* the results are opposite to those of *HGE,* leading to favorable impacts upon all considered variables (positive coefficients and statistically significant), including for the 65+ female health conditions (a coefficient of 0.41, statistically significant, *p* < 0.05), but also life expectancy (*LE*), being similar to the results obtained by Yang et al. [31] for some regions in China.

In the case of the EU-15 panel (Figure 5b), *LP* is also unfavorably influenced by *ER_55_64*, but it is more visible than in the case of the EU-13 panel (a coefficient of −0.292, statistically significant, *p* < 0.001), under the jointly indirect impacts of the selected socio-economic factors. Thereby, positive interplays upon *ER_55_64* are also induced by the educational component, tertiary component (*TE_30_34*), *GERD* expenditure (both of them are significant from the statistical point of view, *p* < 0.01 and *ALMP* (even though not statistically significant). Opposite, negative impacts on *ER_55_64* are induced by increased allocations on passive labor market policies (*PLMP*) (−0.182, statistically significant, *p* < 0.001), *EARN* (−0.309, statistically significant, *p* < 0.01), and *EDU* (although not statistically significant). Thus, we can say that, our first hypothesis, *H_1_: There are significant implications of the employment rate of the population aged 55–64 for labor productivity, more emphasized for developed countries (EU-15) than developing ones (EU-13)*, was supported.

The reasons for diminished labor productivity associated with the employment of people in the 55–64 year age group may be found in a lack of availability of educational and training opportunities, a depreciation of psychological and physiological capacities (as Börsch-Supan and Weiss [64] and Feyrer [65]), and the emergence of new technologies that limit employment opportunities. In this vein, as other authors have stated [32], managers need to sustain the aging of the workforce group (55–64-years-old cohort) through offering opportunities for lifelong learning and training, flexible working arrangements, and health assistance. Employers report a range of factors that favor the hiring of older workers [66]. Bersin and Chamorro-Premuzic [67] argue that employers must take action to “give older people titles and roles that let them contribute their expertise”.

In this regard, the need for reconsidered strategies in both groups of countries is justified, since “less productive older workers should not be considered a reason not to promote the increased participation in the labor force of this population” [23] (p. 94).

Furthermore, under the cascade effects of health expenditure, *HGE* and *HS* (indirect influences), *LP* is positively influenced by almost all variables, more visible for *LE* (a coefficient of 2.252, statistically significant, *p* < 0.01) and *BR* (a coefficient of 0.239, statistically significant, *p* < 0.01). These results are similar to those obtained by Bussolo et al. [26] (p. 113), who argued that “as people live longer they also work until older ages. With slower population growth, it can become easier to increase capital-to-labor ratios and boost worker productivity”. A positive influence on *LP,* in our case, is also obtained by *PGPH* (a coefficient of 0.405, statistically significant, *p* < 0.001) and *HLY_F* (a coefficient of 0.528, statistically significant, *p* < 0.001), being in line with Hansen [47] and Gong, Li, and Wang [46], who argued that healthy individuals are more productive, raising the standard of living for all. The only variable that induced negative impacts upon *LP* under the *HGE* and *HS* implications is *HLY_M* (a coefficient of 0.435, statistically significant, *p* < 0.01). In this situation, further analysis concerning female and male health conditions is required in order to deepen understanding. Therefore, we can say that our third hypothesis, *H_3:_ There are substantial impacts of health expenditure on older people’s health conditions and overall health perceptions, both for the EU-15 and EU-13 panels*, was supported.

As regards health expenditure, in contrast to the EU-13 panel, in the case of the EU-15, *HGE* induced positive effects upon all variables, namely, aging dimensions (*BR* and *LE*) and healthy conditions and perception (*HLY_F*, *HLY_M*, and *PGPH*), since the coefficients for all of these components are positive and statistically significant. The most outstanding favorable impacts of *HGE* are on healthy conditions of women (*HLY_F*) and birth rate (*BR*), which are very significant as regards aging support and positive implications for labor market outcomes, since “the average health conditions of an ageing population play a first-order effect in a macroeconomic perspective, and will represent a key factor for economic growth in the 21st century” [22] (p. 2). As for *HS*, the results are opposite to *HGE* and those obtained within the EU-13 group, with unfavorable effects upon the aging dimensions, health conditions (both women and men) and people’s health perceptions—*PGPH* (negative coefficients and statistically significant). Thus, the second hypothesis, *H_2_: There are significant implications of health expenditure upon aging coordinates (birth rate and life expectancy) in both the EU-15 and EU-13 panels, more distinguished for developed countries (EU-15) than developing ones (EU-13)*, was partially supported.

Ultimately, the fourth hypothesis, H_4_: There are overall (direct, indirect, total) significant implications of health dimensions and aging upon labor productivity, both for the EU-15 and EU-13 panels, was also supported.

In summary, for the EU-13 countries, labor productivity is highly unfavorably influenced by the “share of people with good or very good perceived health”, a very significant SDG target, as regards the health goal. These results bring to the fore several essentials in the case of the EU-13 MS that those concerned with developing labor market policy must consider following the good practice model of the Nordic states [68,69] and, as stressed by Káčerová and Mládek [3] (p. 275), the importance of looking for “opportunities for further utilization of the capabilities and knowledge of the elderly population”: maintaining the 55–64 age group’s skills formation, with particular regards to digital transformation, to which this cohort of people are less adapted than the younger generation [70,71,72]; public health expenditure for further increasing birth rates and sustaining life expectancy, since the highest health needs/consumptions are for the elderly, both women and men; and public health expenditure for increasing the general health condition and perception of people, which would positively influence labor productivity, as argued by Sharpe [23].

For the EU-15 countries, the focus has to be on the following policy interventions: A rethinking of passive labor market policies in order to sustain the 55–65-year-old working group, but also to target increases of active labor market policies for this age cohort; keeping up the R&D expenditure, oriented toward labor skills of the 55–64-year-old working group; reconsideration of *EDU* for 55–64-year-olds, levels 3–8, oriented towards migrants’ inclusion in educational programs (since, in these countries, there is a large flow of migrants from developing countries) [73], and also to 55–64-year-olds for native working people by reshaping their skills as requirements are transformed by the emergence of new digital technologies [68,74]; oriented hospital services to boost healthy conditions and perceptions of people, and thus, making them confident in the public health expenditure in the case of child birth and care, therefore indirectly sustaining birth rates and life expectancy; further government health expenditure aimed at tackling the population aging phenomenon correlated with increasing long-term healthcare in the case of the elderly; and promoting and supporting preventive health actions among people aged 55–64 and low- and medium-educated people, since these categories are the most distant from health prevention, as Bremer et al. [27] demonstrated. Under these cumulative interventions, labor productivity within developed EU MS will increase, with direct effects on wellbeing and overall economic development [10,20,35,36,37,38].

### 4.2. Results of the Gaussian Graphical Models (GGMs)

**Figure 6:**
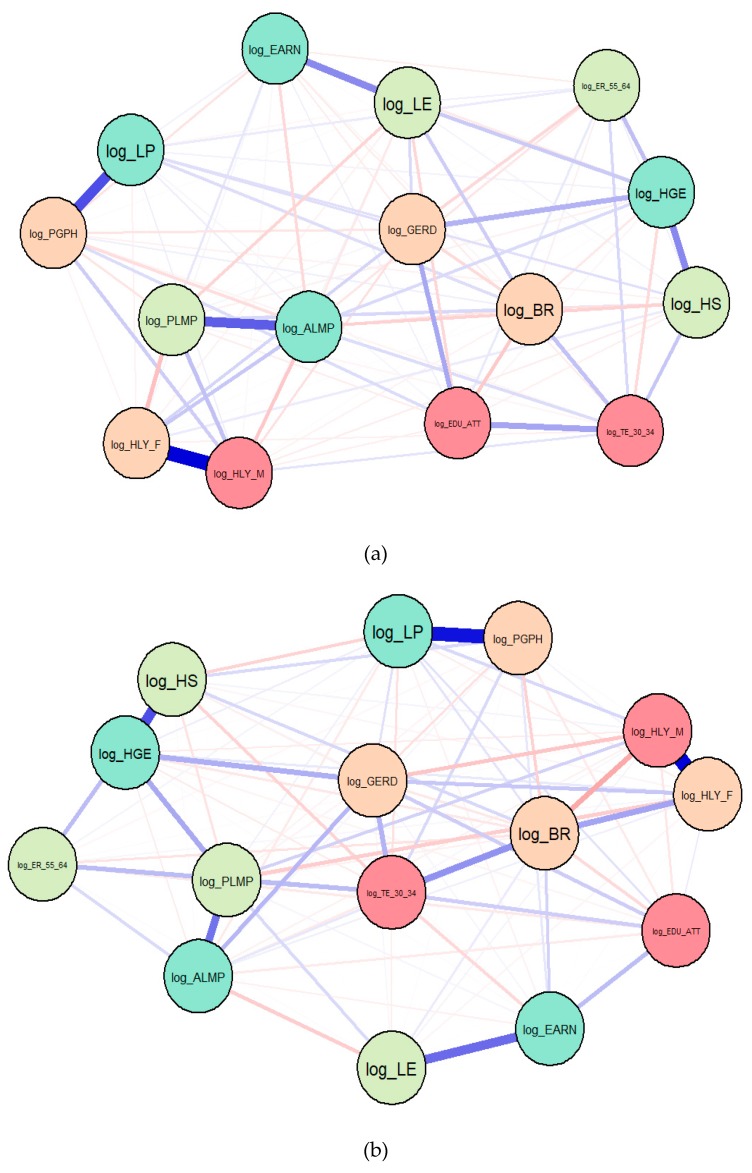
Gaussian graphical models (GGMs), 1995–2017, partial correlation (PCOR): (**a**) EU-13; (**b**) EU-15. Source: Authors’ research in R.

**Figure A1:**
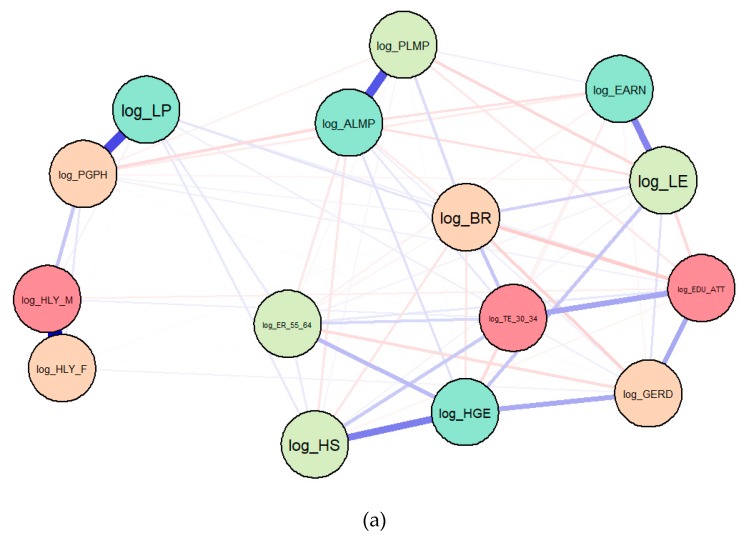

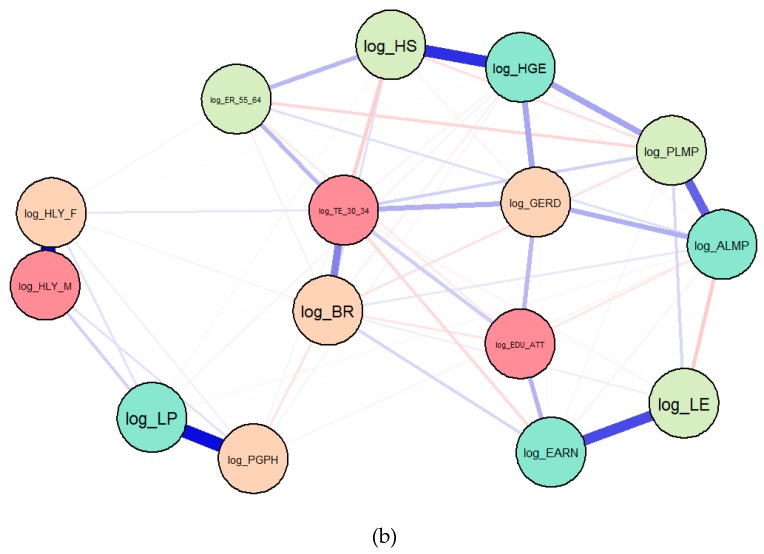
GGMs (Gaussian graphical models), 1995–2017, extended Bayesian information criterion (EBIC), and least absolute shrinkage and selection operator (LASSO): (**a**) EU-13; (**b**) EU-15. Source: Authors’ research in R.

On the back of accurate SEM findings, we continued the research endeavor by designing two sets of Gaussian graphical models (GGMs). These new research settings allowed us to observe the intensity and configuration of the interlinkages between all selected variables/indicators for both EU-13 and EU-15 countries, and to reassess our hypotheses. GGMs are deployed based on partial correlation (PCOR) (Figure 6) and extended Bayesian information criteria (EBIC)/least absolute shrinkage and selection operator (LASSO) (Figure A1 in the Appendix A), separately for the EU-13 and EU-15 countries, for the period 1995–2017. Each indicator previously described in Section 3 (Data and Methodology) represents a node in the configured network, connected with the other coordinates/nodes through significant paths, according to the intensity of the interdependencies between them.

The GGMs (Figure 6) allowed us to obtain new insights into the strong relationships between labor productivity (*LP*) and the share of people with good or very good perceived health (*PGPH*) in the case of both panels of EU-13 and EU-15 countries, which are further linked with health support, namely, healthy life years in absolute value at 65+ for males (*HLY_M*) and females (*HLY_F*) (*reconfirmation of H_3_*) and the employment rate of the population aged 55–64 years (*ER_55_64*) (*reconfirmation of H_1_*), mediated by the life expectancy (*LE*) (*reconfirmation of H_2_*). These results are in line with Hansen [47] and Gong, Li, and Wang [46], who also found that healthy individuals are more productive and generate competitiveness, raising the standard of living for all. In the case of the EU-13 countries, health expenditure and the employment rate of population aged 55–64 years are decisively connected with labor productivity through labor market policies (active and passive) (*ALMP*, *PLMP*) (*reconfirmation of H_4_*). When considering the EU-15 MS, however, these linkages are mediated by birth rate (*BR*), along with educational background (*TE_30_34*), and the expenditure granted for research and development activities (*GERD*).

At the same time, public health expenditure has significant effects on aging coordinates (birth rate and life expectancy) and the employment rate of the population aged 55–64 (*ER_55_64*) in EU-13 countries, as entailed by the strong connections between life expectancy (*LE*), birth rate (*BR*), health government expenditures (*HGE*), and hospital services (*HS*) (*reconfirmation of H_2_*). In EU-15 countries, these linkages and the impact on the labor market integration of older workers (*ER_55_64*) are further enhanced through active and passive labor market policies (*ALMP*, *PLMP*), with positive spillovers for labor productivity (*LP*) (*reconfirmation of H_1_*).

Similar results were obtained when we processed the GGMs through the extended Bayesian information criteria (Figure A1), namely, labor productivity in relation to a proper insertion of persons aged 55–64 into the labor market (a significant active participation of people aged 55–64 years into the labor market reflected through increases in *ER_55_64*) under the decisive contribution of health expenditure (*HS* and *HGE*), leading to an improvement in terms of older people’s health conditions (*HLY_F*, *HLY_M*) and overall population’s health perceptions (*PGPH)*. Hence, the GGMs (Figure 5; Figure A1) reinforce the previous SEM results and confirm the strong interdependences between all considered variables and the significant implications of good public health for labor market performance/outcomes for all EU-28 MS. Thereby, the fourth integrative hypothesis, *H_4_: There are overall (direct, indirect, total) significant implications of health dimensions and aging upon labor productivity, both for the EU-15 and EU-13 panels*, was reconfirmed and supported.

### 4.3. Results of Macroeconometric Models

The results obtained after processing these new sets of macroeconometric models (RREG and PCSE) for each of the four working hypotheses are similar to the SEM model, being robust and consistent throughout different techniques, as follows: unfavorable implications of the proportion of employees aged 55–64 for labor productivity, more emphasized for developed countries (EU-15) than developing ones (EU-13) (H_1_, revealed by the results from Table A6); significant implications of health expenditure upon aging coordinates (birth rate and life expectancy), on the one hand, and on elderly health conditions and overall population health perception, on the other hand, in both EU-15 and EU-13 panels, although more prominent for former (H_2_ and H_3_, disclosed by the results from Table A7); and significant implications of health and aging dimensions upon labor productivity, both for the EU-15 and EU-13 panels (H_4_, as shown by the results from Table A8).

Hence, the macroeconometric models reinforce previous estimations, thus confirming and fulfilling each research hypothesis. Along these lines, this current research also points to many crucial gaps in knowledge and capacity, and strengthens the existing empirical aging literature with an updated complex assessment of the implications of health expenditure upon aging coordinates (birth rate and life expectancy), and further upon the EU labor market’s performance, namely, labor productivity.

Our estimations support several policy interventions that should be implemented by policy-makers across the EU, in about a five-year timeframe, in order to support a decade of concerted actions on the aging phenomenon, so as to strengthen healthy aging, in line with the global strategy of the World Health Organization [42]. Some of these actions include: Additional governmental financial expenditure for health and long-term care systems, since our regression results have shown that an increase in *HGE* leads to significant increases in labor productivity (*LP*); and financial incentives and support services for new mothers to increase birth rates, since our estimations demonstrate that increased birth rates and life expectancy also lead to an increase in labor productivity. Other policy actions should focus on the collection of better global data on healthy aging and, particularly, an accurate dataset at the EU level, promoting research oriented to the needs of older people in order to identify proper means/mechanisms to increase labor productivity and to ensure their adequate labor market integration, reconfiguring health and long-term care systems in every EU Member State (particularly in EU-13 countries) to support the needs of older people and to enhance an age-friendly environment for the community. All of these aspects should be long-term-oriented and generally cover a few decades in order to support active and healthy aging, with benefit spillovers for the wellbeing of older people and the welfare of society as a whole.

## 5. Conclusions

The population aging phenomenon brings with it challenges and opportunities for both developed and developing nations, more emphasized for the EU region than others. The main challenges posed by the increasing share of older individuals are of an economic and social nature, where governments need to cope with increasing expenditures and declining revenues. In this article, we focused on the fact that population ageing tends to equate to increased healthcare expenditure, especially in the case of long-term care needs, with possible implications for labor market outcomes. Thus, the general objective of our paper was to assess the effects of population aging and public health expenditure on labor market performance within the EU-28 MS, with a detailed analysis of the EU-15 (developed countries) and the EU-13 (developing countries) MS.

This research endeavor focused on testing four hypotheses by applying three advanced and integrative econometric techniques, namely, structural equation modeling, Gaussian graphical models (GGMs), and macroeconometric models (robust regression (RREG) and panel corrected standard errors (PCSE)).

After testing the first hypothesis, we found significant implications of the employment rate of the population aged 55–64 for labor productivity, under several social and economic shaping factors, more prominent for developed countries (EU-15), given their higher level of aging dimensions, than for developing ones (EU-13). Special attention, in this regard, for the EU-13 countries must be paid to labor market policies (active and passive), R&D expenditure oriented toward the labor conditions of the workforce aged 55–64, educational support for the 55–64 age group’s skills formation connected to digital transformation. For the EU-15 countries the removal of passive labor market policies is necessary, since *PLMP* are an important factor for labor market insertion, covering mainly unemployment benefits and early retirement schemes that can act as a disincentive for active labor market participation, along with tailored educational programs, to reshape the skills of the workforce aged 55–64.

Further, the second hypothesis assessed significant impacts of health expenditure on aging dimensions in both EU-15 and EU-13 panels, also more distinguished for the former. We found particular directions to be pursued by the EU-13 countries in this vein, namely, on public health expenditure for further encouraging birth rates and sustaining life expectancy. In the case of the EU-15 countries, attention has to be oriented toward hospital services’ contribution to enhance people’s perceptions with regard to public health support, in order to boost the birth rate and life expectancy (aging dimensions). Fundamentally, reshaped policies and strategies embedded in the best practices of EU MS with the highest birth rates are needed (respectively, France, the UK, Sweden, and Austria).

The third hypothesis revealed substantial impacts of health expenditure on elderly health conditions and overall population health perceptions, both for the EU-15 and the EU-13 panels. For the EU-13 countries, we found a lack of public health expenditure, and for the EU-15, of public hospital services. Thus, specific policies have to be made to enhance the overall health perception of people (in EU-13), and to assure better health conditions for people aged 65+ (in EU-15) through the public system.

Finally, under the cumulative interventions, for assessing the fourth hypothesis, when integrative research was applied by SEM and GGMs, we found that *labor productivity is significantly shaped by health dimensions and aging, both for the EU-15 and EU-13 MS*. Within the EU-13, particular focus has to be given to the employment rate of those aged 55–64, people’s health perceptions, and the healthy conditions of women aged 65+. In the case of the EU-15 countries, the focus has to be also on the working labor force aged 55–64 and the health conditions of men aged 65+.

We emphasize that the lack of decisive accurate polices designed to address imbalances will put a strain on public finances, households, and, eventually, lead to lowered productivity and welfare or stagnation. Older individuals who are healthy can support growth through direct or indirect labor force participation or by participating in community programs aimed at assisting other older individuals to cope with the challenges of aging. The first step in improving the health status of a population is to adopt healthy aging mindsets through preventive approaches, with the help of medical professionals and awareness campaigns, especially for the elderly and the segment of the population aged 55–64.

Research limitations reside in the fact that predicting with a high degree of certainty how population aging will affect healthcare costs, and vice versa, is rather difficult, since the aging–health–labor market conjunction is “highly complex and variable”, as Lloyd-Sherlock [59] (p. 887) has emphasized. Aging and health paradigms are influenced by a plethora of factors, hence this research cannot claim to be all-encompassing. Further research will foresee other dimensions related to each country’s economic and social conditions, broken down by countries’ regions (as Yang et al. [31] proposed, taking into consideration, for instance that, capital cities are more developed within each country), but also older people’s gender specificity with regard to their involvement in society [74], as well as related to the aging implications on company risk and return, particularly in emerging markets [75]. A distinct focus of our future research will be on aging policies that need to be tailored to each EU Member State according to their heterogeneity.

## Appendix A

**Table A1:** Summary statistics for the EU-28, 1995–2017.

Variables	*N*	Mean	Sd	Min	Max
**EU-13**					
*LP*	169	70.75858	14.42612	35.7	96.1
*ER_55_64*	261	41.49157	11.0632	17.1	68.1
*GERD*	299	0.8074314	0.4609393	0.024	2.604
*EDU*	299	71.79532	14.59404	17.1	88
*EARN*	273	35414.8	43926.32	666.42	265212
*LE*	273	75.16593	3.220439	67.7	82.7
*BR*	299	10.07358	1.053504	7.6	15.2
*TE_30_34*	299	26.13512	12.49351	1	58.7
*ALMP*	161	0.2024037	0.1598259	0.019	0.873
*PLMP*	162	0.3844074	0.2232345	0.065	1.365
*HLY_F*	161	7.165217	2.504128	2.7	14.2
*HLY_M*	161	7.017391	2.335047	3	13.5
*HGE*	289	4.975779	1.415306	1.8	7.9
*HS*	251	2.669323	0.6839117	0.8	5.1
*PGPH*	164	59.72256	9.925003	35	80.3
*N* total	131				
**EU-15**					
*LP*	195	115.0303	21.86116	75.6	188.2
*ER_55_64*	343	45.74577	12.38773	22.2	76.4
*GERD*	345	1.886067	0.8329314	0	3.914
*EDU*	341	63.13856	13.93617	19.3	82.3
*EARN*	317	46101.44	27095.49	1648.52	311052
*LE*	330	79.63242	1.790216	75.3	83.5
*BR*	345	11.01391	1.720831	7.6	16.7
*TE_30_34*	345	33.01696	11.05093	8.6	54.6
*ALMP*	266	0.6394812	0.3517511	0.034	2.082
*PLMP*	278	1.362842	0.6684728	0.15	3.126
*HLY_F*	192	9.878646	2.337115	5.2	16.8
*HLY_M*	192	9.684375	1.839294	6.2	15.7
*HGE*	345	6.488696	1.085938	3.7	8.9
*HS*	304	3.065461	1.436566	0	6.3
*PGPH*	196	70.79031	8.242352	45.9	84.5
*N* total	146				

Source: Authors’ contribution in Stata.

**Table A2:** Empirical results for SEM models designed to capture total effects of population aging and public healthcare services on labor productivity in the EU, 1995–2017.

Variables	EU-13			EU-15		
	Coef.	Std. err.	*p*-Value	Coef.	Std. err	*p*-Value
Log_ER_55_64 <-						
Log_EARN	−0.044	0.020	0.025	−0.308	0.099	0.002
Log_ALMP	−0.106	0.019	−0.000	0.040	0.033	0.237
Log_PLMP	−0.066	0.259	0.011	−0.181	0.036	0.000
Log_EDU_ATT	0.493	0.079	0.000	−0.135	0.104	0.195
Log_TE_30_34	0.328	0.040	0.000	0.293	0.060	0.000
Log_GERD	−0.037	0.285	0.184	0.314	0.046	0.000
Log_LP <-						
Log_ER_55_64	−0.144	0.050	0.004	−0.292	0.046	0.000
Log_BR	0.105	0.152	0.488	0.239	0.079	0.003
Log_LE	6.366	0.351	0.000	2.251	0.803	0.005
Log_PGPH	−0.585	0.088	0.000	0.404	0.106	0.000
Log_HLY_F	−0.440	0.109	0.000	0.527	0.140	0.000
Log_HLY_M	0.266	0.131	0.043	−0.434	0.155	0.005
Log_EARN	0.006	0.003	0.078	0.090	0.032	0.005
Log_ALMP	0.015	0.006	0.012	−0.011	0.010	0.245
Log_PLMP	0.009	0.005	0.057	0.053	0.013	0.000
Log_DU_ATT	−0.071	0.027	0.010	0.039	0.031	0.204
Log_TE_30_34	−0.047	0.017	0.007	−0.085	0.022	0.000
Log_GERD	0.005	0.004	0.228	−0.091	0.019	0.000
Log_HS	0.247	0.140	0.078	−0.067	0.029	0.021
Log_HGE	−0.001	0.119	0.989	0.277	0.145	0.056
Log_BR <-						
Log_HS	0.096	0.031	0.002	−0.081	0.026	0.002
Log_HGE	−0.095	0.027	0.000	0.371	0.130	0.004
Log_LE <-						
Log_HS	0.069	0.016	0.000	−0.007	0.001	0.000
Log_HGE	−0.023	0.014	0.097	0.033	0.009	0.001
Log_PGPH <-						
Log_HS	0.215	0.073	0.003	−0.028	0.021	0.190
Log_HGE	−0.230	0.062	0.000	0.048	0.108	0.655
Log_HLY_F <-						
Log_HS	0.405	0.158	0.011	−0.095	0.040	0.017
Log_HGE	−0.264	0.135	0.050	0.406	0.200	0.043
Log_HLY_M <-						
Log_HS	0.384	−0.146	0.009	−0.072	0.031	0.021
Log_HGE	−0.354	0.124	0.004	0.277	0.158	0.080

Source: Authors’ contribution in Stata.

**Table A3:** Cronbach’s alpha for the SEM models, EU-13 and EU-15, 1995–2017.

Test Scale = Mean (Standardized Items)								
Average	EU-13				EU-15			
Item	Obs	Sign	Interitem Correlation	Alpha	Obs	Sign	Interitem Correlation	Alpha
Log_ER_55_64	261	−	0.0782	0.5428	343	+	0.1895	0.7660
Log_LP	169	+	0.0577	0.4615	195	+	0.1632	0.7319
Log_BR	299	−	0.0963	0.5988	345	+	0.1848	0.7604
Log_LE	273	+	0.0478	0.4129	330	+	0.1948	0.7720
Log_PGPH	164	+	0.0534	0.4413	196	+	0.1657	0.7355
Log_HLY_F	161	+	0.0502	0.4250	192	+	0.1597	0.7269
Log_HLY_M	161	+	0.0460	0.4029	192	+	0.1642	0.7334
Log_EARN	273	+	0.0614	0.4779	317	+	0.1774	0.7512
Log_ALMP	161	−	0.0812	0.5530	266	+	0.1856	0.7613
Log_PLMP	162	+	0.0726	0.5230	278	+	0.20006	0.7784
Log_EDU_ATT	299	−	0.0694	0.5106	341	+	0.1616	0.7296
Log_TE_30_34	299	+	0.0901	0.5808	345	+	0.1502	0.7121
Log_GERD	299	+	0.0873	0.5725	344	+	0.1724	0.7447
Log_HS	251	+	0.0709	0.5163	287	−	0.1985	0.7762
Log_HGE	289	−	0.1089	0.6310	345	−	0.2389	0.8146
Total scale				0.6346				0.7674

Source: Authors’ contribution in Stata.

**Table A4:** Wald tests for equations associated with the SEM models, EU-13 and EU-15, 1995–2017.

Variables	EU-13			EU-15		
	Chi^2^	df	*p*-Value	Chi^2^	df	*p*-Value
Log_ER_55_64	140.69	6	0.0000	108.28	6	0.0000
Log_LP	456.36	6	0.0000	207.21	6	0.0000
Log_BR	14.34	2	0.0008	10.34	2	0.0057
Log_LE	17.92	2	0.0001	16.34	2	0.0003
Log_PGPH	14.97	2	0.0006	2.37	2	0.3057
Log_HLY_F	7.06	2	0.0293	5.86	2	0.0535
Log_HLY_M	9.84	2	0.0073	5.33	2	0.0695

H0: All coefficients excluding the intercepts are 0. We can thus reject the null hypothesis for each equation, with limitations on PGPH in the case of the EU-15 sample. Source: Authors’ contribution in Stata. df, degrees of freedom.

**Table A5:** Goodness-of-fit tests for the SEM models, EU-13 and EU-15, 1995–2017.

**EU-13**		
Likelihood ratio		
chi2_ms(55)	905.818	model vs. saturated
*p* > chi2	0.000	
chi2_bs(77)	1258.973	baseline vs. saturated
*p* > chi2	0.000	
Information criteria		
AIC	−266.492	Akaike’s information criterion
BIC	−162.984	Bayesian information criterion
Baseline comparison		
CFI	0.280	Comparative fit index
TLI	−0.008	Tucker-Lewis index
Size of residuals		
SRMR	0.070	Standardized root mean squared residual
CD	0.681	Coefficient of determination
**EU-15**		
Likelihood ratio		
chi2_ms(26)	1217.706	model vs. saturated
*p* > chi2	0.000	
chi2_bs(38)	1466.510	baseline vs. saturated
*p* > chi2	0.000	
Information criteria		
AIC	−1259.765	Akaike’s information criterion
BIC	−1152.355	Bayesian information criterion
Baseline comparison		
CFI	0.163	Comparative fit index
TLI	−0.171	Tucker-Lewis index
Size of residuals		
SRMR	0.040	Standardized root mean squared residual
CD	0.550	Coefficient of determination

Source: Authors’ contribution in Stata.

**Table A6:** Results of macroeconometric models deployed for H_1_, EU-13 and EU-15, 1995–2017.

	EU-13		EU-15	
	(1)	(2)	(1)	(2)
	RREG log_LP	PCSE log_LP	RREG log_LP	PCSE log_LP
log_ER_55_64	−0.107 (0.0698)	−0.0918 * (0.0470)	−0.0770 * (0.0365)	−0.127 *** (0.0264)
_cons	4.680 *** (0.265)	4.583 *** (0.175)	5.026 *** (0.142)	5.224 *** (0.103)
*N*	169	169	195	195

Note: Standard errors in parentheses, * *p* < 0.05, ** *p* < 0.01, *** *p* < 0.001. Source: Authors’ contribution in Stata.

**Table A7:** **(a**) Results of macroeconometric models deployed for H_2_ and H_3_, Robust regression (RREG), EU-13 and EU-15, 1995–2017. (**b**) Results of macroeconometric models deployed for H_2_ and H_3_, panel-corrected standard errors (PCSE), EU-13 and EU-15, 1995–2017.

(**a**)					
**EU-13**	**(1)**	**(2)**	**(3)**	**(4)**	**(5)**
	**log_HLY_F**	**log_HLY_M**	**log_BR**	**log_LE**	**log_PGPH**
log_HGE	−0.219 (0.128)	−0.400 ** (0.121)	−0.126 *** (0.0229)	−0.0182 (0.0107)	−0.279 *** (0.0603)
log_HS	0.491 ** (0.150)	0.466 ** (0.142)	0.0952 *** (0.0258)	0.0697 *** (0.0119)	0.235 ** (0.0708)
_cons	1.802 *** (0.179)	2.101 *** (0.169)	2.407 *** (0.0297)	4.286 *** (0.0139)	4.312 *** (0.0826)
*N*	161	161	251	237	164
**EU-15**	**(1)**	**(2)**	**(3)**	**(4)**	**(5)**
	**log_HLY_F**	**log_HLY_M**	**log_BR**	**log_LE**	**log_PGPH**
log_HGE	0.377 * (0.182)	0.245 (0.143)	−0.219 ** (0.0702)	0.102 *** (0.00822)	−0.195 *** (0.0527)
log_HS	−0.0648 (0.0356)	−0.0536 (0.0280)	0.0108 (0.0170)	−0.0172 *** (0.00202)	0.0256 * (0.0103)
_cons	1.606 *** (0.326)	1.832 *** (0.256)	2.805 *** (0.121)	4.204 *** (0.0141)	4.638 *** (0.0944)
*N*	179	179	287	273	182
(**b**)					
**EU-13**	**(1)**	**(2)**	**(3)**	**(4)**	**(5)**
	**log_HLY_F**	**log_HLY_M**	**log_BR**	**log_LE**	**log_PGPH**
log_HGE	−0.301 *** (0.0430)	−0.391 *** (0.0285)	−0.143 *** (0.0137)	−0.0195 *** (0.00538)	−0.255 *** (0.0123)
log_HS	0.493 *** (0.0702)	0.451 *** (0.0605)	0.112 *** (0.0185)	0.0602 *** (0.00682)	0.234 *** (0.0303)
_cons	1.914 *** (0.0563)	2.088 *** (0.0498)	2.422 *** (0.0184)	4.298 *** (0.00787)	4.261 *** (0.0257)
*N*	161	161	251	237	164
**EU-15**	**(1)**	**(2)**	**(3)**	**(4)**	**(5)**
	**log_HLY_F**	**log_HLY_M**	**log_BR**	**log_LE**	**log_PGPH**
log_HGE	0.378 ** (0.142)	0.239 * (0.0949)	−0.219 ** (0.0702)	0.102 *** (0.00822)	0.0219 (0.0598)
log_HS	−0.0690 ** (0.0257)	−0.0547 ** (0.0177)	0.0108 (0.0170)	−0.0172 *** (0.00202)	−0.0194 * (0.00795)
_cons	1.604 *** (0.253)	1.843 *** (0.167)	2.805 *** (0.121)	4.204 *** (0.0141)	4.230 *** (0.108)
*N*	179	179	287	273	182

Note: Standard errors in parentheses, * *p* < 0.05, ** *p* < 0.01, *** *p* < 0.001. Source: Authors’ contribution in Stata.

**Table A8:** Results of macroeconometric models deployed for H_4_, EU-13 and EU-15, 1995–2017.

	EU-13		EU-15	
	(1) RREG	(2) PCSE	(3) RREG	(4) PCSE
	log_LP	log_LP	log_LP	log_LP
log_HGE	0.195 *** (0.0460)	0.174 *** (0.0232)	0.554 *** (0.0684)	0.371 *** (0.0966)
log_HS	−0.178 *** (0.0525)	−0.134 *** (0.0354)	−0.218 *** (0.0135)	−0.189 *** (0.0177)
log_HLY_F	−0.361 ** (0.111)	−0.376 *** (0.0860)	0.357 *** (0.0939)	0.395 *** (0.0821)
log_HLY_M	0.213 (0.135)	0.227 * (0.106)	−0.414 *** (0.107)	−0.443 *** (0.0829)
log_BR	0.205 (0.142)	0.120 (0.158)	0.0462 (0.0563)	0.144 (0.0795)
log_LE	6.112 *** (0.378)	6.113 *** (0.319)	−0.833 (0.577)	0.0420 (0.352)
log_PGPH	−0.417 *** (0.0847)	−0.407 *** (0.0603)	0.564 *** (0.0726)	0.511 *** (0.0576)
_cons	–20.90 *** (1.459)	−20.75 *** (1.078)	5.162 * (2.482)	1.621 (1.436)
*N*	148	148	165	165
*R* ^2^	0.759	0.771	0.797	0.771

Note: Standard errors in parentheses, * *p* < 0.05, ** *p* < 0.01, *** *p* < 0.001. Source: Authors’ contribution in Stata.
